# Mitral valve replacement with annuloplasty in a patient with infected mitral annular calcification

**DOI:** 10.1002/ccr3.7802

**Published:** 2023-08-15

**Authors:** Tatsuya Horibe, Hideaki Hidaka, Ryosuke Numaguchi, Jun Takaki, Kosaku Nishigawa, Takashi Yoshinaga, Toshihiro Fukui

**Affiliations:** ^1^ Department of Cardiovascular Surgery Kumamoto University Hospital Kumamoto Japan

**Keywords:** annuloplasty, infection, mitral annular calcification, mitral valve replacement

## Abstract

**Key Clinical Message:**

Extensive resection of the infected calcified annulus and the reconstruction with a pericardial patch for the debrided annulus is an effective surgical option for the treatment of infectious endocarditis in patients with mitral annular calcification.

**Abstract:**

A 78‐year‐old woman was referred to our hospital because of left‐sided hemiparesis. During the treatment for cerebral infarction, the patient became feverish and lost consciousness. Transthoracic echocardiography revealed mitral annular calcification and a tumor‐like mass on the posterior leaflet despite no findings of mitral regurgitation. She underwent successful mitral valve replacement with debridement of the infected mitral annulus and reconstruction of the posterior annulus (annuloplasty) with bovine pericardium after removal of the mitral annular calcification.

## Graphical abstract

There was an inhomogeneous mass with calcification attached to the posterior annulus, which caused the embolization and it was considered for surgical intervention.

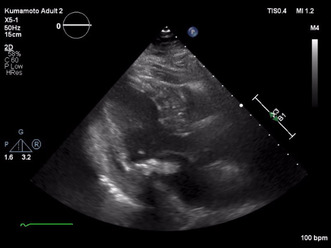

## 1 INTRODUCTION

Mitral annular calcification (MAC) is a chronic degenerative process of the fibrous support structure of the mitral valve.^1^ Its clinical relevance comes from MAC's association with increased rate of mortality and cardiovascular disease.^2^ Stroke and endocarditis are considered to be complications of MAC. Here, we describe mitral valve replacement with debridement of the infected mitral annulus and reconstruction of the posterior annulus (annuloplasty) with bovine pericardium after removal of MAC in a patient with no mitral regurgitation who had a stroke.

## 2 CASE REPORT

**FIGURE 1:**
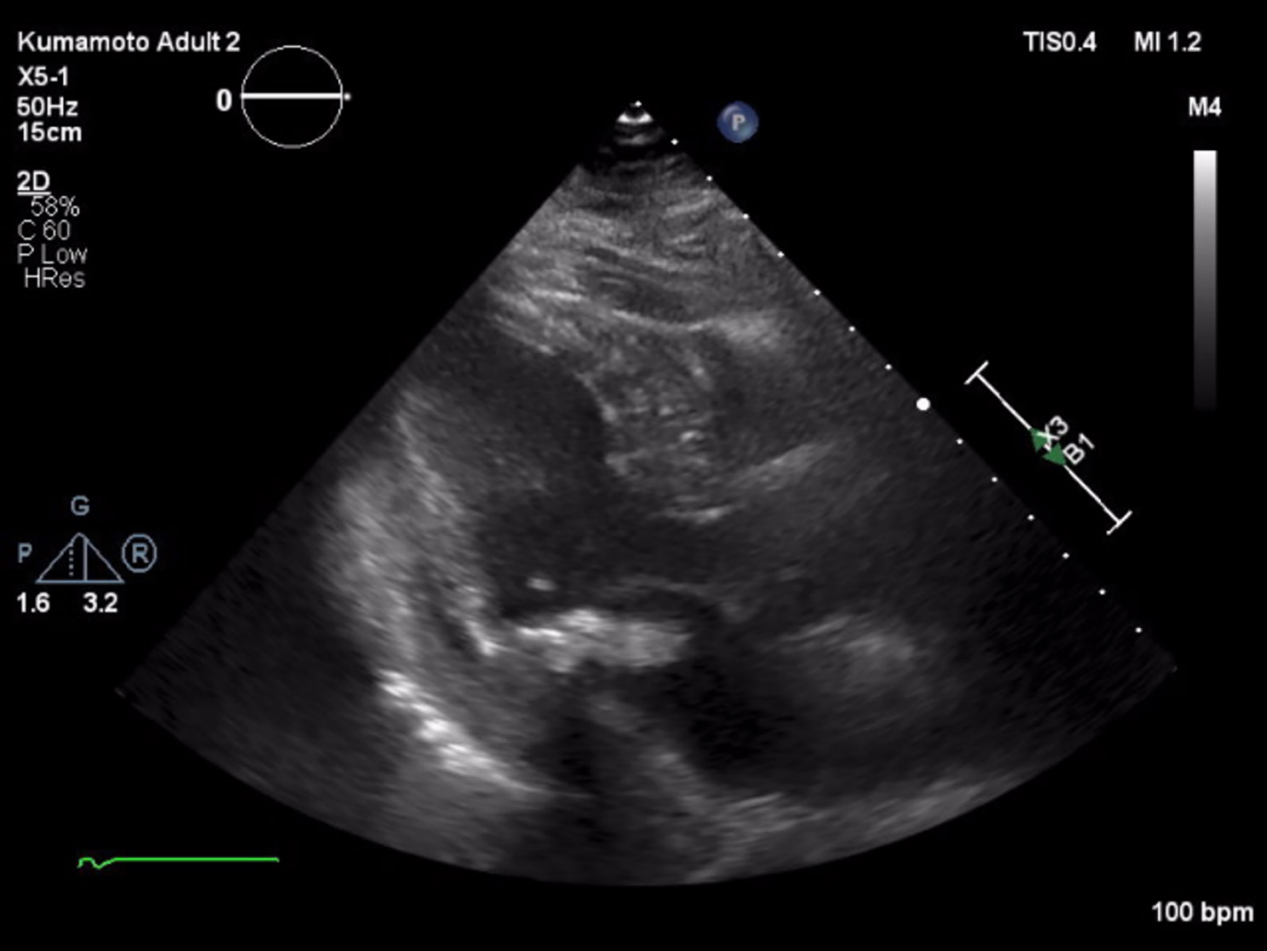
Two‐dimensional transthoracic echocardiography scan shows an inhomogeneous mass attached to posterior annuls with calcification.

**FIGURE 2:**
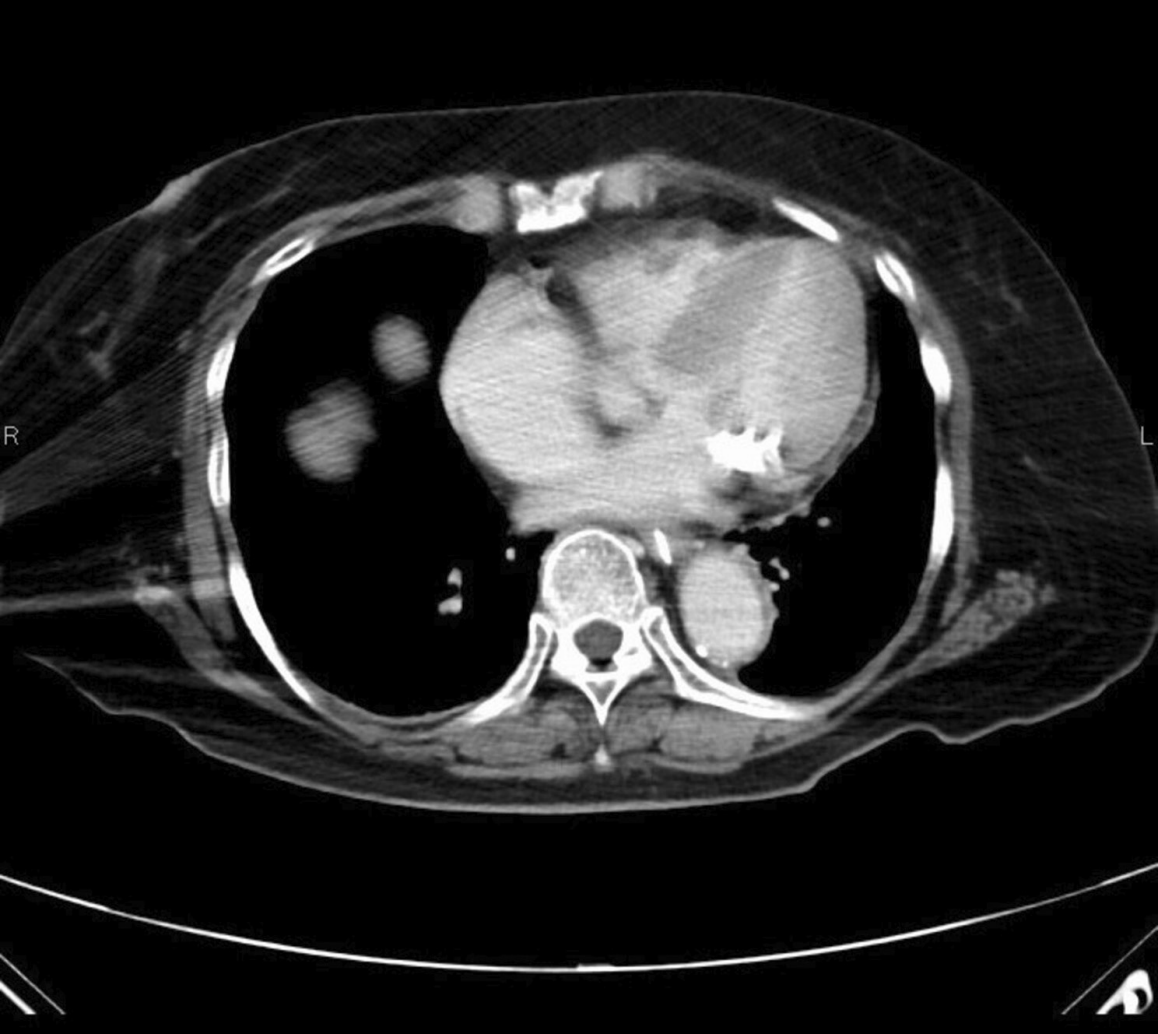
Chest computed tomography shows massive calcification of posterolateral aspect of mitral annulus.

A 78‐year‐old woman was referred to our hospital because of left‐sided hemiparesis. Magnetic resonance imaging (MRI) of the brain revealed multiple cerebral infarctions in the right hemisphere. The source of embolization was atrial fibrillation at that time; therefore, a direct oral anticoagulant was initiated. She had no murmurs on auscultation and was afebrile. On Day 16 of cerebral infarction treatment, she suddenly became feverish and lost consciousness. Antibiotic treatment started 11 days before the surgical treatment. Brain MRI showed only an atypical small cerebral hemorrhage. Transthoracic echocardiography revealed MAC and a tumor‐like mass on the posterior leaflet; however, there was no evidence of mitral stenosis or regurgitation (Figure 1). Because the mass was mobile and 15 mm in size, we planned to perform emergent surgery. Preoperative computed tomography revealed extensive MAC through the posterior mitral annulus (Figure 2).

**FIGURE 3:**
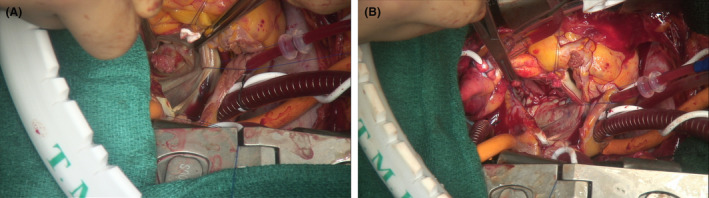
Intraoperative images. (A) Vegetation is attached to the posterior annulus. (B) After extensive debridement, mitral annulus was repaired with bovine pericardium.

Cardiopulmonary bypass was performed through ascending aortic cannulation and bicaval drainage. Cardiac arrest was achieved with aortic cross‐clamping and the administration of blood cardioplegia. Mitral valve exposure was achieved through a superior transseptal approach. A friable mass was observed on the posterior annulus, which included a severely calcified bar (Figure 3A). The mass was easily excised. The anterior and posterior leaflets were completely resected, and the calcified posterior annulus was carefully decalcified using scissors, rongeurs, and Cavitron ultrasonic surgical aspirator (CUSA EXcel; Integra Life Sciences). The procedure was performed according to a previously reported manner.^3^ Decalcification was performed until all calcium was removed and circumscribing atrioventricular fat or ventricular myocardium was identified. One of the margins of the pericardial patch was sutured to the posterior left atrial wall, and the other was sutured to the smooth endocardium of the healthy LV myocardium using a continuous 4–0 polypropylene suture to cover the entire debrided area (Figure 3B). The bioprosthetic valve was implanted in the supra‐annular position using non‐everted horizontal mattress sutures. The patient was successfully weaned off cardiopulmonary bypass with intra‐aortic balloon pumping (IABP), which was prophylactically placed to avoid left ventricular rupture. The IABP was removed on postoperative Day 5, and the patient was extubated on Day 6. The histopathological examination of the removed 15 mm diameter mobile mass showed acute and chronic vegetation inflammation which is indicative of infective endocarditis. Postoperative echocardiography demonstrated no pseudoaneurysm of the left ventricle and no paravalvular mitral regurgitation. Transthoracic echocardiography at 8 months postoperatively showed left ventricular ejection fraction was 67%, no paravalvular leakage, Vmax was 1.6 m/s, meanPG was 5.6 mmHg, and PHT was 72 ms.

## 3 DISCUSSION

MAC is known to be associated with cardiovascular events and mortality. The Framingham Heart Study demonstrated that MAC was associated with an increased risk of incident cardiovascular disease (hazard ratio [HR] 1.5), cardiovascular mortality (HR 1.6), and all‐cause mortality (HR 1.3).^2^ MAC has several cardiac implications, such as arrhythmia, mitral valve regurgitation, mitral valve stenosis, and endocarditis. However, the risk of endocarditis in patients with MAC remains unknown.^1^ In a review of 80 patients with MAC, Fulkerson et al. reported three cases of endocarditis (4%).^4^ Endocarditis lesions include vegetation on the mitral valve leaflets and, less commonly, on the calcified annulus as well as paravalvular abscesses in the MAC region.^5^ Poor clinical outcomes and increased mortality were associated with mitral annulus lesions rather than leaflet lesions. Limited data also suggest high mortality (29%) among patients with MAC undergoing surgery for MR due to infective endocarditis.^6^ Some reports have described perivalvular abscess or pseudoaneurysm formation around MAC. Ozawa et al. reported an autopsy case of perivalvular abscess superimposed on MAC, which penetrated the pericardial cavity.^7^ Kim et al. reported a case of endocarditis with a subannular pseudoaneurysm along the MAC, which was repaired surgically.^8^ Therefore, early diagnosis and treatment are crucial for patients with MAC who are diagnosed with endocarditis. In our case, the patient was diagnosed with infectious endocarditis because of persistent fever after the hospitalization for cerebral infarction, although she did not have mitral valve stenosis or regurgitation. Surgery could then be planned immediately after the diagnosis.

There are several surgical treatment methods for patients with MAC, including mitral valve surgery (repair or replacement), with or without annular calcium debridement, and edge‐to‐edge repair. It is generally considered that debridement of the MAC may lead to cardiac rupture at the atrioventricular junction and circumflex artery injury. However, some surgeons consider that complete resection of debris and reconstruction of the annulus are essential for the treatment of endocarditis in patients with MAC.^9^ We believe that infected leaflets and calcified annuls should be removed as much as possible to prevent recurrence of infection and reconstruct the annuls with pericardium in order to secure the prosthetic valve and prevent left ventricular rupture. Extensive resection of infected calcified annulus may cause serious complications such as cardiac rupture, left circumflex coronary artery injury. To prevent left ventricular rupture, we utilized the IABP. Bavaria and associates have proven a statistically significant reduction in left ventricle peak pressure and left ventricle end diastolic pressure with the use of IABP in their animal studies.^10^ The IABP will be beneficial to decrease the afterload and help to prevent excessive buildup of intraventricular pressure. It will decrease the tension along the repaired suture line and avoid the stitches cutting and left ventricle rupture after mitral valve replacement.^11^ We believe that using an IABP can effectively prevent left ventricle rupture after mitral valve replacement during the postoperative period, especially when there is a risk.

## 4 CONCLUSIONS

MAC was the most conceivable cause of endocarditis and cerebral infarction in this case. Extensive resection of infected calcified annulus is a surgical option for the treatment of infectious endocarditis in patients with MAC. And the postoperative use of the IABP is a considerable option for the prevention of left ventricle rupture after extensive resection of infected calcified annulus.

## AUTHOR CONTRIBUTIONS

**Tatsuya Horibe:** Writing – original draft. **Hideaki Hidaka:** Writing – review and editing. **Ryosuke Numaguchi:** Writing – review and editing. **Jun Takaki:** Writing – review and editing. **Kosaku Nishigawa:** Writing – review and editing. **Takashi Yoshinaga:** Writing – review and editing. **Toshihiro Fukui:** Writing – review and editing.

## FUNDING INFORMATION

The authors report no involvement in the research by the sponsor that could have influenced the outcome of this work.

## CONFLICT OF INTEREST STATEMENT

The authors certify that there is no conflict of interest with any financial organization regarding the material discussed in the manuscript.

## CONSENT STATEMENT

Written informed consent was obtained from the patient to publish this report in accordance with the journal's patient consent policy.

## Data Availability

The data that support the findings of this study are available from the corresponding author, T.H, upon reasonable request.
